# Contagious(?) Keratitis in the Cow

**Published:** 1881-10

**Authors:** Wm. H. Arrowsmith

**Affiliations:** 3 Wayne St., Jersey City


### IV.—CONTAGIOUS (?) KERATITIS IN THE COW.

EDITOR OF JOURNAL OF COMPARATIVE MEDICINE:

In compliance with your request I send you this report.

Was called to examine the cattle of a dairyman at Bergen Hill, N. J., which were suffering from an apparently infectious disease of the eyes.

Two weeks before he had purchased a cow, which had been brought from the upper part of New York State. Was in good health, and about twelve years of age. Three days after the purchase he noticed a slight watery effusion, and two days later a small grayish disk appeared on the cornea just over the pupil of the left eye, and continued to grow larger. Still he felt no concern till he noticed the same symptoms in the eyes of one of his own cows, and two days later another (a heifer) showed the same signs. It was at this point that I saw the cattle.

The eyes of the first cow were totally destroyed. The grayish disk had extended over one-third of the cornea. The fibrous membrane of the eyelids, conjunctiva, etc., were suppurating or greatly congested. Four other cows were affected, and showed the different stages of the disease as follows:

A lachrymal effusion only in an animal which had had the disease during two days.

The same effusion and a grayish disk in a case of five days' standing.

In the other two, of ten and twelve days' duration, the disk had enlarged and a partial suppuration taken place.

All were affected in the left eye first, and the disease continued more severe in that than in the right.

I gave an antiseptic of boracic acid in the first case, in connection with belladonna, and to the other four, careful washing with cold water, belladonna solution being injected with syringe.

The eyes of the first cow are totally destroyed. They are still greatly congested, and are apparently being absorbed. The animal is, however, in good health.

The next two cattle affected can still see, and their eyes are slowly recovering their normal condition.

The symptoms of the two last affected have entirely disappeared; but in all the small white disk still remains upon the cornea.

No other cattle of the herd have been affected, and those affected were all in perfectly good health.

Can we consider this a case of malignant inflammation of the cornea transmitted by actual contact, or is it a new disease of the eyes—infectious and perhaps parasitical—similar to that which has lately affected the cattle in certain districts of the West?

WM. H. ARROWSMITH, D.V.S.

3 WAYNE ST., Jersey City.

